# An overview on the current available treatment for COVID-19 and the impact of antibiotic administration during the pandemic

**DOI:** 10.1590/1414-431X2021e11631

**Published:** 2021-12-10

**Authors:** H.S.C. Paula, S.B. Santiago, L.A. Araújo, C.F. Pedroso, T.A. Marinho, I.A.J. Gonçalves, T.A.P. Santos, R.S. Pinheiro, G.A. Oliveira, K.A. Batista

**Affiliations:** 1 Departamento de Áreas Acadêmicas, Instituto Federal de Educação, Ciência e Tecnologia de Goiás, Campus Goiânia Oeste, Goiânia, GO, Brasil; 2 Instituto Federal de Educação, Ciência e Tecnologia de Goiás, Campus Valparaíso, Valparaíso, GO, Brasil

**Keywords:** COVID-19, SARS-CoV-2, Antimicrobial resistance, Multidrug-resistant bacteria

## Abstract

Severe acute respiratory syndrome coronavirus 2 (SARS-CoV-2) infection has caused several problems in healthcare systems around the world, as to date, there is no effective and specific treatment against all forms of COVID-19. Currently, drugs with therapeutic potential are being tested, including antiviral, anti-inflammatory, anti-malarial, immunotherapy, and antibiotics. Although antibiotics have no direct effect on viral infections, they are often used against secondary bacterial infections, or even as empiric treatment to reduce viral load, infection, and replication of coronaviruses. However, there are many concerns about this therapeutic approach as it may accelerate and/or increase the long-term rates of antimicrobial resistance (AMR). We focused this overview on exploring candidate drugs for COVID-19 therapy, including antibiotics, considering the lack of specific treatment and that it is unclear whether the widespread use of antibiotics in the treatment of COVID-19 has implications for the emergence and transmission of multidrug-resistant bacteria.

## Introduction

In recent decades, the emergence of severe acute respiratory syndromes related to coronaviruses, namely MERS-CoV, SARS-CoV, and SARS-CoV-2 (COVID-19), has triggered unprecedented responses to control the infection and protect those most vulnerable, with special emphasis on the global pandemic status of COVID-19. Since its outbreak in Wuhan, in December 2019, the ongoing COVID-19 pandemic has affected (as of May 16, 2021) 162,184,263 people and caused 3,364,446 deaths (1), becoming the most significant health concern of present times. On March 2021, Brazil became the epicenter of COVID-19 pandemic, reaching more then 400,000 deaths by the end of April 2021. Efforts have been made worldwide to rapidly develop COVID-19 vaccines, and to date, there are more than two hundred candidate vaccines, nine of which are currently approved in various countries (2). The Brazilian National Health Surveillance Agency (ANVISA) has approved four vaccines to date, three of which are for emergency use only and one developed by Pfizer-BioNTech has received final approval. However, there are no known specific, effective, and proven therapies against COVID-19. Remdesivir, an antiviral agent, is currently one of the few drugs approved by the Food and Drug Administration (FDA-USA) and by the ANVISA, specifically for the treatment of COVID-19.

The pandemic has led to the emergence of drug repositioning as a short-term strategy to yield a treatment for COVID-19, and the use of antimicrobial drugs in clinical trials as a potential direct therapy to treat and prevent COVID-19 has increased (3-6). In addition, the rapid increase in antibiotic use, particularly in hospitals, could exert a strong selective pressure on bacteria to develop resistance. This could contribute to an increase in the emergence of multidrug-resistant organisms (MDRO) in the months and years following the end of the pandemic.

In this scenario, it is important to consider the short- and long-term consequences of the current COVID-19 pandemic on drug-resistant infections, especially in the acute-care setting, as patients are exposed to increased antimicrobials that are often used suboptimally or inappropriately (7,8). The possibility of an increase in antimicrobial resistance (AMR) following a pandemic is a problem that has serious implications for global health, affecting both treatment costs and morbidity and mortality rates (9). Therefore, in light of the current COVID-19 pandemic, this overview discusses drug treatment strategies, focusing on antimicrobial therapy and the short- and long-term consequences of its administration on the development of AMR.

## Current therapeutic options for COVID-19

The global outbreaks of SARS in 2003 (∼8500 individuals infected, 10% fatality rate), MERS-CoV in 2012 (∼2500 individuals infected, 35% fatality rate), and the recent COVID-19 pandemic have demonstrated the need to investigate treatment options for severe coronavirus infections (10). Recently, with the outbreak of COVID-19, one by one new therapeutic strategies are emerging (Table 1), depending on the severity of disease development and patient comorbidities.

**Table 1:** Current therapeutic options for COVID-19.

Therapy	Mechanism of action	Approval for COVID-19 use	Recommendations	Reference
Colchicine	Anti-mitotic, anti-inflammatory activities. It interferes with secretion of inflammatory cytokines.	Not approved	There is insufficient evidence to recommend the use for the treatment of COVID-19.	(14,15)
Chloroquine and Hydroxychloroquine	The drugs seem to interfere with the activity of ACE2, making it unable to interact with the SARS-CoV-2 spike protein, preventing the virus from entering the cells. The drugs interfere with the endosomal pH, which may affect the ability of the virus to fuse and enter cells.	Not approved	Not recommended for prophylactic use nor for treatment of COVID-19 infected individuals independent of illness severity.	(37,39)
Dexamethasone	Blockade of inflammation pathways; vasodilation and immune cell migration.	FDA ANVISA	Recommended for hospitalized patients requiring supplemental oxygen or mechanical ventilation.	(18)
Remdesivir	Inhibitor of viral RNA polymerase.	FDA ANVISA	Recommended for hospitalized patients requiring supplemental oxygen, but not recommended for routine use in patients who require mechanical ventilation.	(34)
Convalescent plasma	Passive immunity in the form of neutralizing antibodies obtained using apheresis in survivors with prior infection of SARS-CoV-2.	FDA ANVISA	Recommended only in the context of a clinical trial.	(22,25)
Monoclonal antibodies				
Bamlanivimab and Etesevimab	Bamlanivimab and etesevimab bind to different but overlapping sites on the receptor binding domain of the spike protein of SARS-CoV-2 blocking its attachment to the human ACE2 receptor.	FDA ANVISA	Recommended for the treatment of mild to moderate COVID-19 in adults and children over 12 years old and weighing at least 40 kg.	(30,31)
Tocilizumab	Tocilizumab is a recombinant humanized anti-IL-6 receptor monoclonal antibody that inhibits the binding of IL-6 to both membrane and soluble IL-6 receptors, blocking IL-6 signaling and reducing inflammation.	FDA	Suggested use on hospitalized with severe or critical disease receiving systemic corticosteroids and requiring supplemental oxygen or mechanical ventilation.	(32)
Casirivimab and Imdevimab	Casirivimab and imdevimab bind to different sites on the receptor binding domain of the spike protein of SARS-CoV-2, blocking its attachment to the human ACE2 receptor.	FDA ANVISA	Patients with mild to moderate COVID-19 at high risk of progression to severe disease admitted to the hospital for reasons other than COVID-19.	(33)

FDA: Food and Drug Administration (USA); ANVISA: Brazilian National Health Surveillance Agency (Brazil).

For non-hospitalized patients with mild COVID-19 symptoms, the treatment consists of medicines to alleviate and/or ameliorate symptoms (i.e., cough, nasal congestion, dyspnea, diarrhea, and fever) combined with hydration, nutritional supplementation and, in some cases, antibiotic therapy. General treatments based on nutritional interventions and the use of immune-enhancers were also supposed to boost the immune system to fight SARS-CoV, MERS-CoV, and SARS-CoV-2 (11). Probiotic supplementation has been suggested as a possible alternative to modulate the immune system and improve outcomes, such as ventilator-associated pneumonia in patients hospitalized with COVID-19. In addition, mineral and vitamin supplementation can be very important to improve the immune system response to coronavirus infections (11,12). Another therapeutic approach to increase the immune response is the use of interferon-α (IFN-α), an immune-enhancer that interferes with the replication of the coronavirus viral genomes by stimulating the innate and adaptive immune responses (13).

### Colchicine

For decades, colchicine has been successfully used for the treatment of auto-immune and inflammatory disorders. Colchicine is an old, inexpensive drug that has anti-mitotic and anti-inflammatory activities and inhibits the secretion of inflammatory cytokines. Due to its anti-inflammatory effect, colchicine has been proposed to have a beneficial effect on COVID-19 clinical outcomes, reducing severity, and mortality rates (14,15). A Brazilian single-center randomized, double-blinded, placebo controlled clinical trial showed that the use of colchicine reduced the length of both supplemental oxygen therapy and hospitalization in moderate to severe COVID-19 patients. The drug was safe and well tolerated (16). However, studies with a larger number of patients should be conducted to confirm the efficacy and safety of colchicine for COVID-19 treatment.

### Dexamethasone

Severe COVID-19 can lead to inflammatory organ injury due to the occurrence of a macrophage activation syndrome and production of excessive pro-inflammatory molecules, with elevated plasma levels of inflammatory markers, including C-reactive protein, ferritin, interleukin-1, and interleukin-6 (17). The excessive and uncontrolled production of soluble inflammatory markers, known as ‘cytokine storm', is a major cause of acute respiratory distress syndrome in COVID-19 patients, a primary cause of mortality (5). Several therapeutic interventions have been proposed to mitigate the inflammatory organ injury caused by viral infections. Dexamethasone, a synthetic glucocorticoid, has been shown to improve survival of severe hospitalized COVID-19 patients who require supplemental oxygen, with the greatest benefit observed in patients who require mechanical ventilation (18).

Dexamethasone acts by blocking inflammatory pathways: vasodilation and immune cell migration. After crossing the host cell membrane, dexamethasone binds to cytoplasm receptors and initiates a cascade of responses that leads to suppression of the following pro-inflammatory cytokines: interleukin (IL)-1, IL-2, IL-6, IL-8, tumor necrosis factor (TNF), and interferon (IFN)-γ, and increases gene expression of IL-10, an anti-inflammatory cytokine mediator, as well as prevents chemotaxis at the site of inflammation, and inhibits the activation of macrophages (19). Corticosteroids such as dexamethasone are usually safe drugs for short-term use, despite the potential for adverse effects with prolonged administration. Prolonged use may be associated with glaucoma, cataract, fluid retention, temporary hyperglycemia, hypertension, weight gain, increased risk of infections, and osteoporosis (20). The American Center for Disease Control and Prevention (CDC, USA) recommends the use of dexamethasone for hospitalized patients with COVID-19 who require supplemental oxygen delivery through a high-flow device, noninvasive ventilation, invasive mechanical ventilation, or extracorporeal membrane oxygenation (ECMO) (21).

### Immunotherapy

Convalescent plasma therapy is a strategy of passive immunotherapy that has often been used in COVID-19 patients. The strategy consists of using apheresis in survivors with prior infection of SARS-COV-2 to obtain neutralizing antibodies that target and eradicate the pathogen on the recipient. However, in addition to neutralizing antibodies, other proteins such as anti-inflammatory cytokines, clotting factors, natural antibodies, defensins, pentraxins, and other undefined proteins are obtained from donors. Thus, convalescent plasma could provide further benefits such as immunomodulation through the amelioration of severe inflammatory response, and could later interfere with the cytokine storm, common in severe cases of COVID-19 (22). However, a recent study showed that anti-IFN-α antibodies were confirmed in COVID-19 patients (23), and these autoantibodies could worsen the effect of any viral infection and were recently associated with a high risk of developing life-threatening pneumonia in COVID-19 patients (24). Although many observational studies have shown that convalescent plasma therapy has some curative effect and is well tolerated in the treatment of infectious diseases (25), a recent systematic review and meta-analysis demonstrated that convalescent plasma has minimal or no benefit in the treatment of COVID-19 (26). For critically ill patients, data are still controversial. While some studies provide evidence of reduced length of hospital stay, disease severity, and mortality, with a low frequency of adverse events in a considerable number of patients, other studies have failed to find evidence of benefit for convalescent plasma administration (27-29). Therefore, passive immunotherapy with convalescent plasma for COVID-19 patients is inconclusive and needs to be validated in large controlled clinical trials. However, another type of immunotherapy using monoclonal antibodies (bamlanivimab, etesevimab, tocilizumab, casirivimab, and imdevimab) has recently been approved by the FDA and ANVISA. These types of monoclonal antibodies seem to be effective against the S protein of SARS-CoV-2 in the treatment of mild to moderate Covid-19 forms (30-33).

### Remdesivir

Antiviral drugs have been considered potential candidates and have been evaluated regarding their effectiveness against SARS-CoV-2. For severe cases of COVID-19, remdesivir is the only antiviral agent approved by the FDA and currently recommended for use in hospitalized patients who require supplemental oxygen. However, its routine use in patients who require mechanical ventilation is not recommended due to the lack of data showing benefit in this advanced stage of the disease. In Brazil, remdesivir was the first drug approved for the treatment of COVID-19 (ANVISA March/2021). Given the high mortality of COVID-19 despite the use of remdesivir, treatment with an antiviral drug alone is unlikely to be sufficient for all patients (34). Remdesivir, which acts as a nucleoside analog, is a broad-spectrum antiviral drug that is effective against MERS-CoV and was identified early as a promising candidate for COVID-19 treatment (35) due to its ability to inhibit SARS-CoV-2 at low concentrations *in vitro* (4). However, a recent study, the WHO SOLIDARITY trial, evaluated the effect of four repurposed antiviral drugs, including remdesivir, and results showed that remdesivir has little effect on hospitalized patients with COVID-19 (36). These unpromising overall findings refute early hopes that the use of remdesivir alone would substantially reduce inpatient mortality, initiation of ventilation, and hospitalization duration.

### Chloroquine and hydroxychloroquine

Chloroquine (CQ) and hydroxychloroquine (HCQ) are antimalarial drugs that are indicated as treatment against SARS-CoV (37) and MERS-CoV (38), and were tested as antiviral treatment for COVID-19. CQ and HQC apparently act by interfering with the activity of angiotensin-converting enzyme 2 (ACE2), making it unable to interact with the SARS-CoV-2 spike protein, preventing the virus from entering the cells (37). In addition, CQ and HQC seem to interfere with the endosomal pH, affecting the ability of the virus to fuse and enter cells (39). Early in the COVID-19 pandemic, studies reported that the use of CQ/HCQ at the beginning of the disease was beneficial (40-42). However, conflicting results about the efficacy and safety of these anti-malarial drugs have emerged, and the WHO has discouraged the use of CQ/HCQ for the treatment of COVID-19. The HCQ arm of the SOLIDARITY trial showed that HCQ has no effect on the mortality of hospitalized patients with COVID-19 compared to standard care (35). These findings were also reported in the trial study developed by the RECOVERY Collaborative Group (43), with the results showing that patients who received HCQ do not have lower mortality than those who receive the usual care. While the effects of CQ/HCQ in the treatment of patients with COVID-19 remain controversial (6,44), special attention must be drawn to the complications associated with the use of these drugs. Antimalarial drugs are associated with various side effects such as cardiomyopathy and QT interval prolongation, which reduces their potential usefulness in some COVID-19 patients, especially those with cardiac comorbidities (45). Considering the risks and benefits of HCQ, the Guideline Development Group (GDG) of WHO published an Interim Guidance on March 2, 2021 (46) strongly advising against the prophylactic use of HCQ in individuals who do not have COVID-19, emphasizing that there is no justification for specifically recommending HCQ for individuals with known exposure to a person with SARS-CoV2 infection (47). A recent meta-analysis evaluated 11,932 COVID-19 patients taking HCQ and 8,081 taking HCQ with azithromycin. The study suggests that HCQ alone was not associated with reduced mortality in hospitalized COVID-19 patients but the combination of HCQ and azithromycin significantly increased mortality (48). Although many Brazilian clinicians defend the so-called “early treatment” of COVID-19, the TOGETHER randomized clinical trial conducted in Brazil recently showed that neither HCQ nor lopinavir-ritonavir had any significant benefit for decreasing COVID-19-associated hospitalizations or other secondary clinical outcomes (49).

### Antibiotic therapy for COVID-19

Considering the possibility of bacterial co-infection in patients with moderate-to-severe COVID-19, antibiotic treatment has often been prescribed (Table 2). There are several studies that present potential benefits of macrolide antibiotic therapy in coronavirus infections because of their immunomodulatory effects and antiviral activities (50-52). Currently, the empirical use of broad-spectrum antibiotics in patients with COVID-19 appears to be common, even without the presence of signs and symptoms of a bacterial coinfection. According to Langford et al. (53), three-quarters of the patients with COVID-19 receive antibiotics, and prescribing is significantly higher than the estimated prevalence of bacterial co-infection. In general, the most commonly used class of antibiotics for COVID-19 patients includes fluoroquinolones, followed by cephalosporins and macrolides, which mainly include azithromycin (54,55). It is possible that these classes of antibiotics are selected for their lung coverage for pneumococcal, gram negative, and atypical bacterial infections.

**Table 2:** Antibiotics with possible use in COVID-19 management.

Antibiotic	Antiviral mechanism	Compliance with WHO guidelines (46)	Reference
Fluoroquinolones		WHO does not recommend the use of antibiotics in mild-to-moderate COVID-19 patients. WHO does not encourage the use of broad-spectrum antibiotics for COVID-19 management, especially those from the Watch and Reserve list. WHO recommends the use of antibiotic therapy for patients with suspected or confirmed severe COVID-19 presenting highly suspected pneumonia based on clinical signs.	(81)
Levofloxacin	Membrane fusion inhibition; replication inhibition; immunomodulatory effects		
Moxifloxacin	Inhibition of SARS-CoV-2 replication; immunomodulatory activity		
Ciprofloxacin	Inhibition of viral replication; immunomodulatory activity.		
Cephalosporines		WHO does not recommend antibiotics for therapy and/or prophylaxis in suspected or confirmed mild-to-moderate COVID-19 patients unless a bacterial infection is confirmed. WHO does not encourage the use of broad-spectrum antibiotics for COVID-19 management, especially second-to-fourth generation cephalosporins, which are listed as Watch and Reserve antibiotics.	(54,55)
First- to fourth-generation	Viral replication inhibition; inhibition of the interaction between the viral spike protein and ACE2 receptors.		
Macrolides		WHO does not recommend antibiotics for therapy and/or prophylaxis in suspected or confirmed mild-to-moderate COVID-19 patients unless a bacterial infection is confirmed; WHO does not recommend the therapeutic and/or prophylactic use of azithromycin alone or with hydroxychloroquine/chloroquine for COVID-19; WHO does not encourage the use of broad-spectrum antibiotics for COVID-19 management, especially those from the Watch and Reserve list.	(57,59,64-67)
Azithromycin	Attenuation of viral infection by (i) increasing endosomal/lysosomal pH; (ii) membrane fusion inhibition; and (iii) amplifying the interferon-mediated antiviral responses; Decreases the production of cytokines, reducing the occurrence of a “cytokine storm”.		
Clarithromycin	Immunomodulatory effects by (i) suppressing the infection-related inflammation and (ii) reducing the vascular hyper-permeability.		
Tetracyclines		WHO recommends the use of antibiotic therapy for patients with suspected or confirmed severe COVID-19 presenting highly suspected pneumonia based on clinical signs. WHO recommends against the use of antibiotic therapy or prophylaxis for patients with suspected or confirmed mild-to-moderate COVID-19 without a presence of a bacterial co-infection. WHO does not encourage the use of broad-spectrum antibiotics for COVID-19 management, especially those from the Watch and Reserve list.	(56)
Doxycycline	Inhibition of viral replication; interference with protein synthesis; immunomodulatory effects.		
Minocycline	Inhibition of protein synthesis; Inhibition of RNA replication; immunomodulatory effects.		

The most commonly used antibiotics with possible antiviral properties are tetracyclines and azithromycin. Tetracyclines, such as doxycycline or minocycline, act by inhibiting protein synthesis and have the advantage of being inexpensive and have a good record, especially for short-term use (56). For patients who cannot tolerate tetracycline derivatives, there is the option of replacing them with macrolides such as clarithromycin or azithromycin (57). Azithromycin is a broad-spectrum macrolide antibiotic that is bacteriostatic against many Gram-positive and Gram-negative bacteria and has few side effects. It is inexpensive and can be rapidly produced on a large-scale, if necessary (58).

One possible antiviral mechanism of azithromycin is its intracellular sequestration that would result in an increase in endosomal and/or lysosomal pH, thereby attenuating viral replication (59). This is a similar mechanism to that proposed for the antiviral effects of CQ and HCQ (60) and could explain why the two drugs, which are both weak bases, can act in a complementary manner to inhibit viral replication (61). Studies on the effectiveness of these drugs are still divergent. The group of Gautret et al. (41) have identified clinical improvement in patients who received HCQ and azithromycin, with a reduction in nasopharyngeal viral load and shorter hospital stays. In contrast, Mehra et al. (62) showed that the combination of these two drugs was associated with an increased risk for clinically significant occurrence of ventricular arrhythmias.

Additionally, the use of azithromycin in combination with quinolone and high doses of corticosteroids for treatment of atypical pneumonia has also been discussed, with an improvement in the clinical picture due to a reduction in acute respiratory distress syndrome and ventilatory support, as well as a lower mortality rate (63). However, there are insufficient clinical data to support an effective use of azithromycin for the treatment of pneumonia as a result of SARS-CoV-2 viral infection.

Azithromycin also has an immunomodulatory ability, being able to induce a global amplification of interferon-mediated antiviral responses, leading to a reduced replication of invading respiratory viruses (64). Recently, Ulrich et al. (65) highlighted that the protein receptor CD147, considered a novel route for SARS-CoV-2 invasion, could be a target for COVID-19 treatment, since azithromycin is supposed to limit endocytosis in macrophages via inhibition of CD147. This inhibition pathway of SARS-CoV-2 viral invasion via CD147 may contribute to positive clinical outcomes of azithromycin treatment for COVID-19. However, further studies need to be carried out to confirm the possible role and the mechanism of azithromycin in SARS-COV-2 infection.

It is known that infection by SARS-CoV and SARS-CoV-2 can lead to hyperinflammation due to the occurrence of a macrophage activation syndrome and production of excessive pro-inflammatory molecules. In this case, the use of macrolides like azithromycin could attenuate excessive cytokine production during coronavirus infection, being effective in reducing TNF-α-stimulated activation of nuclear factor-kappa B (NF-κB), suppression of the synthesis of NF-κB-dependent pro-inflammatory cytokines IL-6 and IL-8 in tracheal aspirate cells, and a decrease in the content of IL-1β produced by macrophages (66,67).

In addition to the possible antiviral effect, azithromycin acts by decreasing mucus secretion by bronchial epithelial cells, facilitating lung function. It is considered a senolytic agent, since it can selectively target and remove senescent cells, with an efficiency of almost 97%, inducing myofibroblast autophagy (61,68). However, blind and inappropriate use of antibiotics, especially broad-spectrum antibiotics, should be avoided. The WHO interim guideline on the clinical management of COVID-19 (69) does not recommend the use of antibiotics for therapy or prophylactic treatment of patients with suspected or confirmed mild to moderate COVID-19 symptoms unless a bacterial infection is confirmed.

Antibiotics like linezolid and vancomycin can augment cytokine production (TNF-α, IL-1β, IL-6, IL-10) by activating Toll-like receptors (TLRs), being associated with the possible pathophysiology of antibiotic-induced septic shock in COVID-19 patients. Although the exact pathophysiology of septic shock in these patients is still unclear, if antibiotics are administered in the absence of bacterial infection, a cytokine storm may initiate with unregulated release of TLRs, IL-1β, IL-6, TNF-α, and gut endotoxins (70,71).

Although antibiotic therapy has been used as an alternative treatment for COVID-19 even in the absence of a bacterial co-infection (53), there are many concerns about the possibility that this therapy may accelerate and/or increase AMR.

## Short- and long-term potential impact of antibiotic use for the treatment of COVID-19

To date, it is difficult to predict how the COVID-19 pandemic might affect antimicrobial stewardship and promote long-term antimicrobial resistance, as data are still insufficient. However, some authors argue that there are still good reasons to treat SARS-CoV-2 with antibiotics, even in the absence of a bacterial co-infection, to avoid serious complications from viral infections (41,56,72-74).

Although there are many reports of bacterial co-infections in diseases caused by SARS-CoV-2 (75-77), the unnecessary use of antibiotics is likely to be high in patients with COVID-19 (53). A recent review of 19 studies showed that the minority of patients, about 17.6%, receiving antibiotics were shown to have secondary or bacterial co-infection, indicating a major empiric use of antibiotics (54).

The association between COVID-19 and the occurrence of healthcare-associated superinfections can be explained by two non-exclusive possibilities. First, severe infections with SARS-CoV-2 result in dysregulation of the immune system, making patients more vulnerable to bacterial proliferation (78). Patients with severe COVID-19 present lower levels of CD4^+^ and CD8^+^ lymphocytes, decreased interferon-gamma expression by CD4^+^ cells, and a higher level of pro- and anti-inflammatory cytokines, which may predispose them to superinfections (78,79). Second, critically ill patients, independent of COVID-19, who require intensive care or mechanical ventilation, have a markedly increased risk of bacterial infections.

In a pandemic context, most actions and interventions are empirical and based on preclinical studies, *in vitro* experiments, and observational studies without adequate methodology. Considering this scenario, the Brazilian COVID-19 Guidelines for Pharmacological Treatment recommend avoiding the inappropriate use of broad-spectrum antibacterial drugs and not using prophylactic antibacterial drugs in patients with suspected or diagnosed COVID-19 without the presence of a bacterial co-infection (80). If the clinical symptoms suggest a bacterial infection, patients with mild symptoms can take antibiotics for community-acquired pneumonia, such as amoxicillin, azithromycin, or fluoroquinolones (i.e., levofloxacin, moxifloxacin, ciprofloxacin), and with severe symptoms, empirical treatment should cover all possible pathogens (81).

Growing evidence suggests that a considerably high proportion of COVID-19 patients are being unnecessarily treated with antibiotics. In a rapid review and meta-analyses, Langford et al. (53) found that 75% of patients with COVID-19 received antibiotics, although a low rate of co-infection was reported in these patients. In patients from Asia, Rawson et al. (82) reported that empirical antibiotics were prescribed for 72% of patients despite low confirmation of secondary bacterial infections (<10%). In addition, it has been reported that about 90% of patients with COVID-19 are being unnecessarily treated with antibiotics and 98% of these prescriptions were empiric (83). In addition, although this was not measured, it is possible that large numbers of people self-medicate with antibiotics to protect themselves from the virus. This self-medication may actually be very common in developing countries where antibiotics can be easily obtained without a prescription. In all these cases, the overuse of antibiotics has been shown to be a risk factor for increased AMR and the development of superinfections in acute-care settings and also in the community (84).

Regarding the pressure on healthcare systems caused by the COVID-19 pandemic, it is likely that the redistribution of health professionals, overwork, and focus directed at SARS-COV-2 have led to neglect of the management of antibiotic prescriptions and prevention of the spread of multidrug resistant microorganisms (82). A busy healthcare facility may not be able to monitor antibiotic prescribing and administration as well as in pre-pandemic times. In addition, medical centers are overcrowded and have 50-70% more patients than normal, making it difficult to manage prophylactic and preventive actions to prevent the spread of MRD organisms.

There are many other important factors of antimicrobial resistance that have arisen in this time of pandemics, such as inadequate COVID-19 testing and lack of proper diagnosis of co-infection. Laboratory diagnosis is essential and help in choosing the right treatment. The lack of specific therapy to fight coronaviruses and the rise of many experimental studies with efficient results is also a trigger for inappropriate use of drugs, especially antibiotics, increasing multidrug resistance of bacteria that can be potentially fatal in coinfections with COVID-19. Overuse of antibiotics can also reduce pharmacy stocks and these drugs may be lacking for the treatment of patients who really need this type of medication (13). Therefore, the global threat of antimicrobial resistance may accelerate as a result of the use of inappropriate antibiotic therapy in COVID-19 patients. In this scenario, health and social systems must act together to mitigate the potential short- and long-term impact of COVID-19 on antimicrobial use and AMR.

## Conclusion and future perspectives based on the current management of COVID-19

The global health emergency of COVID-19 is linked to serious problems such as morbidity, mortality, and economic crises. Regarding the vaccine, efforts are focused on immunization against SARS-CoV-2. On a cautiously optimistic note, immunization combined with appropriate precautions (social distancing, use of masks, and hygiene measures) could help prevent the spread of SARS-CoV-2. Although the Brazilian Health System (SUS) is considered a world reference in vaccination, COVID-19 immunization is still slow due to the lack of available doses, and Brazil has become a worldwide concern about disease spread and appearance of new variants of the virus.

The pandemic triggered by SARS-CoV-2 posed major challenges to public health and to drug research laboratories. These areas are fundamental to containing the spread of the virus and optimizing the treatment of patients with COVID-19. Despite the short time of in-depth scientific studies on SARS-CoV-2, the sequencing of the complete viral genome showed strong homology with another coronavirus implicated in outbreaks, i.e., SARS-CoV. Therefore, assessing the impact of previous coronaviruses outbreaks and the current impact of COVID-19 could guide decision-making and action in this pandemic, especially regarding the timely development of efficient treatment and vaccines.

Previous drug research efforts have also been useful in the development of broad-spectrum anti-coronaviral agents for the current pandemic. The lack of efficient and specific treatments to stop the progression of the pandemic has demonstrated that drug repurposing seems to be the most efficient tool in the search for a therapeutic solution. The approved repurposing of drugs and experimental drugs with known safety profiles can provide an important reservoir of compounds that can be accelerated for clinical development. However, to date, there is no specific drug for the treatment of COVID-19. According to a recent report by the Brazilian National Commission for the Incorporation of Technologies in SUS (Comissão Nacional de Incorporação de Tecnologias no SUS - Conitec), drugs such as HCQ or CQ, azithromycin, lopinavir/ritonavir, colchicine, and convalescent plasma should not be used in hospitalized patients, because they have been tested and have not shown clinical benefit in this population (85).

In the short-term, antibiotics have played several roles in the SARS and MERS outbreaks, and now in the COVID-19 pandemic (56,65). However, the lack of rapid diagnostic and decision-making tools for the clinical management of COVID-19 has contributed to an increase in unnecessary use of antibiotics. With this in mind, the widespread use of antibiotics should be discouraged, as this may lead to higher rates of bacterial resistance, which will impact the burden of disease and death in the population during the COVID-19 pandemic and beyond.

As antibiotics use increased, bacteria responded by developing various forms of resistance to these treatments, reducing the effectiveness of the drug and limiting the treatment choices against these drug-resistant organisms (86). Indeed, the impact of AMR on patient morbidity and/or mortality poses a growing threat to human health, with significant implications for the global economy and security. The CDC estimates that more than 2.8 million people in the United States acquire health-care-associated superinfections due to MDR organisms each year, and at least 35,000 people die as a consequence of these infections (87). Furthermore, recent data from the Interagency Coordination Group (IAGC) on Antimicrobial Resistance indicate that AMR diseases are responsible for at least 700,000 annual deaths worldwide, and this figure could increase to as many as 10 million deaths per year by 2050 if sustained efforts to contain antimicrobial resistance are not undertaken (88).

The outbreak and subsequent COVID-19 pandemic caused a collapse in healthcare systems worldwide, requiring unprecedented measures to control the spread of infection and treat those infected. To this end, considerable efforts were aimed at improving knowledge about the importance of personal hygiene, environmental contamination, and the use of personal protective equipment. However, the high number of COVID-19 infected individuals who required medical attention and hospital care has driven the need to relax measures that prevent the spread of MDRO, such as screening, isolation in single rooms, and antimicrobial stewardship. Associated with this, the need to redeploy antibiotic stewardship teams and increase laboratory capacity to support the workload associated with COVID-19 diagnosis and management could also contribute to an exacerbation of the number of healthcare-associated superinfections. On the other hand, increased awareness of personal hygiene with an emphasis on hand hygiene, efforts to reduce and/or limit patient contact, and increased social distancing measures can also help reduce the healthcare-associated transmission of AMR and diseases that would drive antibiotic use.

The development of new digital technologies to monitor antimicrobial prescribing based on antibiogram results and institutional multi-resistance profiles may also be useful in controlling the spread of multidrug-resistant bacteria. These novel technologies could be extremely helpful, but their implementation can face difficulties, especially in developing countries, where many hospitals lack the minimum structure for digital technologies, such as electronic medical records. It is known that coordinated data collection and reporting by local facilities can support clinical, epidemiological, and administrative needs. For this chain to transmit timely, reliable, and valid data in a cost-effective manner, an intricate and extensive set of links need to be ensured (89). A digital system using multiple data sources (e.g., CT reports, microbiology results, antimicrobial drug dispensing) could be a promising approach for continuous prospective surveillance, not only for bacteria but also for other microorganisms, especially in the event of a pandemic (90).

Finally, the development of clinical treatment guidelines to limit unnecessary antimicrobial exposure, measures for routine surveillance of AMR, and the review of national policies that do not neglect essential public health programs to control other diseases are essential. In addition, new approaches to antimicrobial stewardship must become an essential part of clinical practice to promote wider adoption and interventions aimed at optimizing treatment and reducing inappropriate antimicrobial use.
